# Blood long non‐coding RNA intersectin 1–2 is highly expressed and links with increased Th17 cells, inflammation, multiple organ dysfunction, and mortality risk in sepsis patients

**DOI:** 10.1002/jcla.24330

**Published:** 2022-03-04

**Authors:** Qinghe Huang, Yibin Wang, Fuyun He

**Affiliations:** ^1^ Department of Central Intensive Care Unit Zhongshan Hospital Affiliated to Xiamen University Xiamen China

**Keywords:** inflammation, lncRNA ITSN1‐2, multiple organ dysfunction, sepsis, Th1 and Th17 cells

## Abstract

**Background:**

Long non‐coding RNA intersectin 1–2 (lnc‐ITSN1‐2) exacerbates inflammation and promotes T‐helper (Th) cell differentiation, also serves as a biomarker in critical illness diseases. However, its clinical role in sepsis remains obscure. Hence, the study aimed to explore the relationship of lnc‐ITSN1‐2 with Th cells, inflammation, disease severity, multiple organ dysfunction, and mortality risk in sepsis.

**Methods:**

Peripheral blood mononuclear cells (PBMC) were isolated from 95 sepsis patients and 50 health controls, followed by lnc‐ITSN1‐2 evaluation using RT‐qPCR. PBMC Th1, Th17 cells and their secreted cytokines in serum were detected by flow cytometry and ELISA, respectively.

**Results:**

Lnc‐ITSN1‐2 in sepsis patients was higher than it in health controls (*Z *= −7.328, *p *< 0.001). Lnc‐ITSN1‐2 correlated with increased interferon‐gamma (*p *= 0.009), Th17 cells (*p *= 0.022), and interleukin‐17A (*p *= 0.006), but not Th1 cells (*p *= 0.169) in sepsis patients. Moreover, lnc‐ITSN1‐2 had a positive connection with C‐reactive protein (*p *= 0.001), acute pathologic and chronic health evaluation (APACHE) II (*p *= 0.024), and sequential organ failure assessment (SOFA) scores (*p *= 0.022). Regarding SOFA subscales, lnc‐ITSN1‐2 linked with elevated respiratory system score (*p *= 0.005), cardiovascular system score (*p *= 0.007), and renal system score (*p *= 0.004) but no other subscales. Besides, lnc‐ITSN1‐2 had an increasing trend, but no statistical difference, in septic deaths compared to survivors (*Z *= −1.852, *p *= 0.064).

**Conclusion:**

Lnc‐ITSN1‐2 reflects sepsis progression and unfavorable prognosis to some extent, which may serve as a potential biomarker to improve the management of sepsis patients.

## Graphical abstract

Long non‐coding RNA intersectin 1‐2 (lnc‐ITSN1‐2) exacerbates inflammation and promotes T‐helper (Th) cell differentiation, also serves as biomarker in critical illness diseases. However, its clinical role in sepsis remains obscure. The findings of this study indicates that lnc‐ITSN1‐2 reflects sepsis progression and unfavorable prognosis to some extent, which may serve as a potential biomarker to improve the management of sepsis patients.

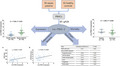

## 1 INTRODUCTION

Sepsis is featured as an uncontrolled host response to infection, which causes approximately 30 million new cases and around 5.3 million deaths every year.^1^, ^2^, ^3^, ^4^ Meanwhile, the pathogenesis of sepsis is complex, including systemic inflammatory response, immune disorder, endoplasmic reticulum stress, and other pathophysiological processes, eventually causing multiple organ dysfunction and leading to high mortality.^5^, ^6^, ^7^ Apart from the above understanding of the pathogenesis of sepsis, several treatment methods have been established for sepsis patients including antibiotics, hemodynamic management, infection source control, etc.; however, their mortality risk remains high.^8^, ^9^, ^10^, ^11^, ^12^ Thus, it is helpful to explore potential biomarkers for sepsis, based on which clinicians could manage patients timely to improve their prognosis.

Long non‐coding RNAs (lncRNAs) are involved in different cellular functions, biologic processes and participate in diverse critical illnesses including cancer, severe acute pancreatitis (SAP), and sepsis.^13^, ^14^, ^15^, ^16^, ^17^ LncRNA intersectin 1–2 (lnc‐ITSN1‐2) is located on chromosome 21 with a length of 451 bp, which is implicated in the mediation of T‐helper (Th) cells, a crucial modulator of inflammation.^16^, ^18^, ^19^ In vitro studies present that lnc‐ITSN1‐2 activates CD4^+^ T cell, then stimulates Th1 and Th17 cell differentiation through targeting microRNA 125a (miR‐125a) in inflammatory bowel disease (IBD)^18^; suppressing lnc‐ITSN1‐2 could reduce inflammation through nucleotide‐binding oligomerization domain 2 (NOD2)/receptor‐interacting protein 2 (RIP2) signaling pathway in rheumatoid arthritis (RA).^20^, ^21^ In clinical studies, lnc‐ITSN1‐2 is dysregulated in critical illnesses such as SAP and acute ischemic stroke (AIS).^16^, ^22^ Besides, prior research illustrates that lnc‐ITSN1‐2 is linked with aggravated inflammation in sepsis patients.^23^ However, the linkage of lnc‐ITSN1‐2 with Th cells and multiple organ dysfunction in sepsis patients remains unknown, which needs to be further explored.

In the current study, we aimed to evaluate the relationship of lnc‐ITSN1‐2 with Th1 cells, Th17 cells, inflammation, multiple organ injury, and 28‐day mortality in sepsis patients.

## 2 METHODS

### 2.1 Participants

From January 2019 to February 2021, this study consecutively recruited 95 sepsis patients. The recruitment criteria were set as (i) diagnosis of sepsis in terms of the sepsis‐3 criteria (2016 version)^24^; (b) aged more than 18 years; (c) hospitalized for sepsis treatment within 24 h of symptom onset. The sepsis patients were excluded from the study if they met the following conditions: (i) unwilling to provide peripheral blood (PB) samples for study use; (ii) complicated with autoimmune disease, solid tumor, or hematologic malignancy; (iii) pregnant and lactating woman. In addition, during the same period, this study also recruited 50 healthy participants with matched age and gender to sepsis patients as health controls. The exclusion criteria for sepsis patients were appropriate for health controls, and the health controls who had abnormalities in the physical examination were also ineligible for the study. The Ethics Committee of Zhongshan Hospital Affiliated to Xiamen University approved the study.

### 2.2 Data collection

Clinical characteristics were collected after admission within 24 h for further analysis, including age, gender, body mass index (BMI), smoke status, drink status, comorbidities, primary infection site, primary organism, C‐reactive protein (CRP) level, Acute Physiology and Chronic Health Evaluation II (APACHE II) score, and Sequential Organ Failure Assessment (SOFA) score. Additionally, the sepsis patients were closely followed up until death or up to 28 days, and mortality during 28‐day follow‐up was recorded.

### 2.3 Sample collection and assessment

PB samples of all sepsis patients were collected after admission, then peripheral blood mononuclear cells (PBMCs) and serum were separated. PBMCs of all sepsis patients were separated to detect lnc‐ITSN1‐2 by reverse transcription‐quantitative polymerase chain reaction (RT‐qPCR). Besides, among 60 sepsis patients, PBMCs were separated to assess the percentage of Th1 and Th17 cells (CD4+ T cells were considered as a denominator in the calculation) by flow cytometry (FCM) using Human Cell Differentiation Kit (Bio‐Techne China Co. Ltd.). Serum samples were separated to detect the level of interferon‐gamma (IFN‐γ) and interleukin‐17A (IL‐17A) by enzyme‐linked immunosorbent assay (ELISA) using commercial Human ELISA Kit (Bio‐Techne China Co. Ltd.). In addition, PB samples of health controls were collected after recruitment, then PBMCs were isolated to evaluate lnc‐ITSN1‐2 by RT‐qPCR. The procedures were in strict accordance with the direction of instructions.

### 2.4 RT‐qPCR assay

The RT‐qPCR assay was performed for determining the lnc‐ITSN1‐2 in PBMCs. In brief, total RNA was extracted by PureZOL RNA isolation reagent (Bio‐Rad); then, was reversely transcribed to cDNA using iScript^™^ cDNA Synthesis Kit (with random primer) (Bio‐Rad); besides, the qPCR was executed by SYBR^®^ Green Real‐time PCR Master Mix (Toyobo). The primers were designed according to a previous study.^21^ Subsequently, the lnc‐ITSN1‐2 was analyzed using the 2^−ΔΔCt^ method (GAPDH as an internal control).

### 2.5 Statistics

Graphics were plotted using GraphPad Prism 7.02 (GraphPad Software Inc.), and statistical analyses were completed using SPSS 24.0 (IBM). Difference of lnc‐ITSN1‐2 between sepsis patients and health controls was compared by Wilcoxon's rank‐sum test. Association of two variables was analyzed by Spearman's rank correlation test. Correlation of lnc‐ITSN1‐2 with primary infection site and primary organism was determined by the Kruskal‐Wallis H rank‐sum test or Wilcoxon's rank‐sum test. Comparison of lnc‐ITSN1‐2, Th1 cells, IFN‐γ level, Th17 cells and IL‐17A level between deaths and survivors were evaluated by Wilcoxon's rank‐sum test. Statistical significance was concluded if a *p*‐value less than 0.05.

## 3 RESULTS

### 3.1 Sepsis patients’ characteristics

Sepsis patients presented a mean age of 56.8 ± 11.8 years with 33 (34.7%) females and 62 (65.3%) males. Moreover, the median (interquartile range (IQR)) values of CRP level was 87.5 (44.3–127.0) mg/L. The APACHE II and SOFA scores were 11.9 ± 5.9 and 5.2 ± 2.4, respectively. Other clinical properties about patients were displayed in Table 1.

**TABLE 1:** Clinical characteristics of sepsis patients

Items	Patients (*N* = 95)
Age (years), mean ± SD	56.8 ± 11.8
Gender, No. (%)	
Female	33 (34.7)
Male	62 (65.3)
BMI (kg/m^2^), mean ± SD	23.4 ± 3.7
Smoke, No. (%)	38 (40.0)
Drink, No. (%)	38 (40.0)
History of hypertension, No. (%)	36 (37.9)
History of hyperlipidemia, No. (%)	16 (16.8)
History of diabetes, No. (%)	15 (15.8)
History of CKD, No. (%)	8 (8.4)
History of CCVD, No. (%)	23 (24.2)
Primary infection site, No. (%)	
Abdominal infection	32 (33.7)
Respiratory infection	25 (26.3)
Skin and soft tissue infection	23 (24.2)
Other infections	15 (15.8)
Primary organism, No. (%)	
G‐ bacteria	54 (56.8)
G+ bacteria	29 (30.5)
Fungus	12 (12.6)
Others	17 (17.9)
Culture negative	14 (14.7)
CRP (mg/L), median (IQR)	87.5 (44.3–127.0)
APACHE II score, mean ± SD	11.9 ± 5.9
SOFA score, mean ± SD	5.2 ± 2.4

Abbreviations: APACHE II, Acute Physiology and Chronic Health Evaluation II; BMI, body mass index; CCVD, cerebrovascular and cardiovascular diseases; CKD, chronic kidney disease; CRP, C‐reactive protein; IQR, interquartile range; SD, standard deviation; SOFA, Sequential Organ Failure Assessment.

### 3.2 Comparison of Lnc‐ITSN1‐2 between sepsis patients with health controls

**FIGURE 1:**
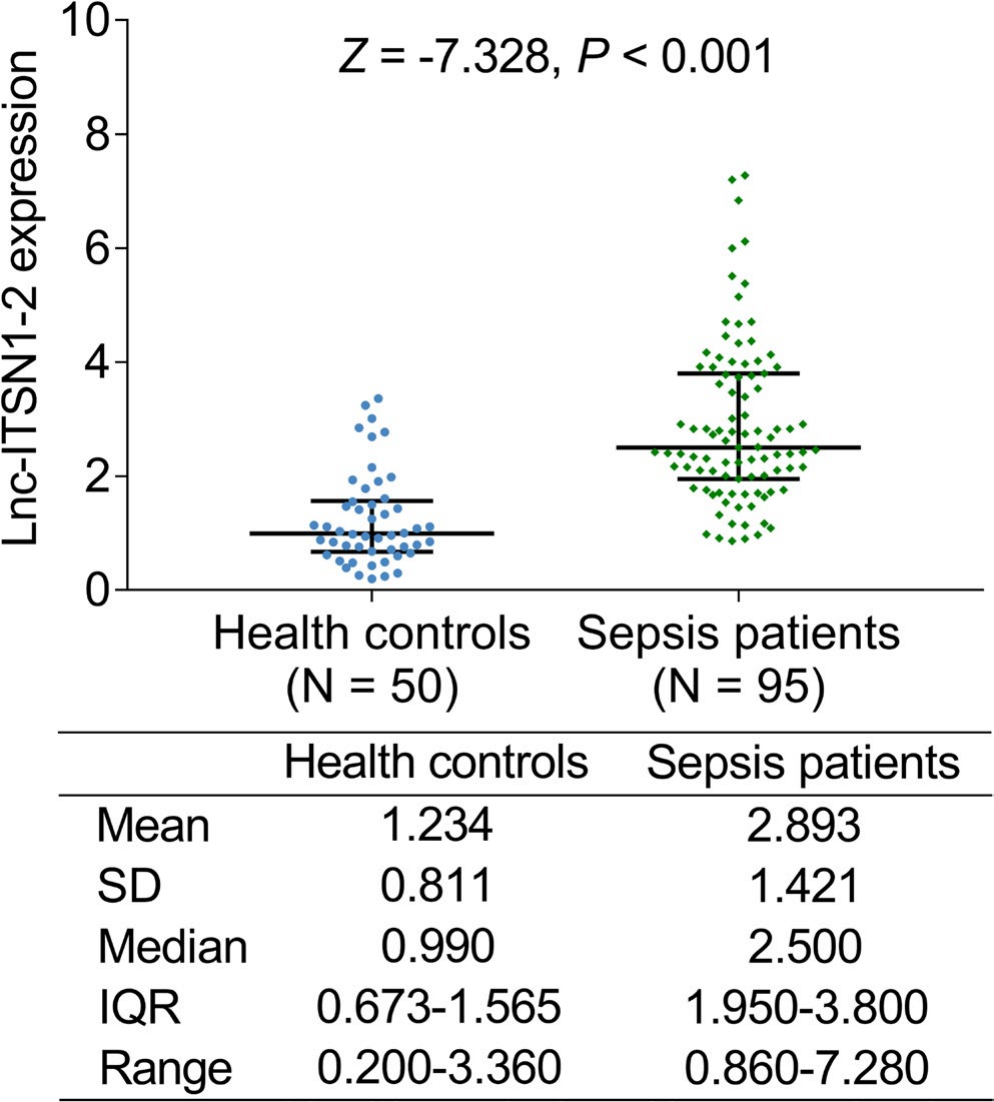
Comparison of lnc‐ITSN1‐2 between health controls and sepsis patients

Lnc‐ITSN1‐2 in sepsis was more increased than it in health controls (*Z *= −7.328, *p *< 0.001) (Figure 1). The mean ± SD value of lnc‐ITSN1‐2 was 2.893 ± 1.421 and 1.234 ± 0.811 in sepsis patients and health controls, respectively; the lnc‐ITSN1‐2 was 2.500 (IQR: 1.950–3.800) in sepsis patients and 0.990 (IQR: 0.673–1.565) in health controls; the range of lnc‐ITSN1‐2 was 0.860–7.280 in sepsis patients and 0.200–3.360 in health controls.

### 3.3 Linkage of lnc‐ITSN1‐2 with Th1 cells, IFN‐γ, Th17 cells, and IL‐17A

**FIGURE 2:**
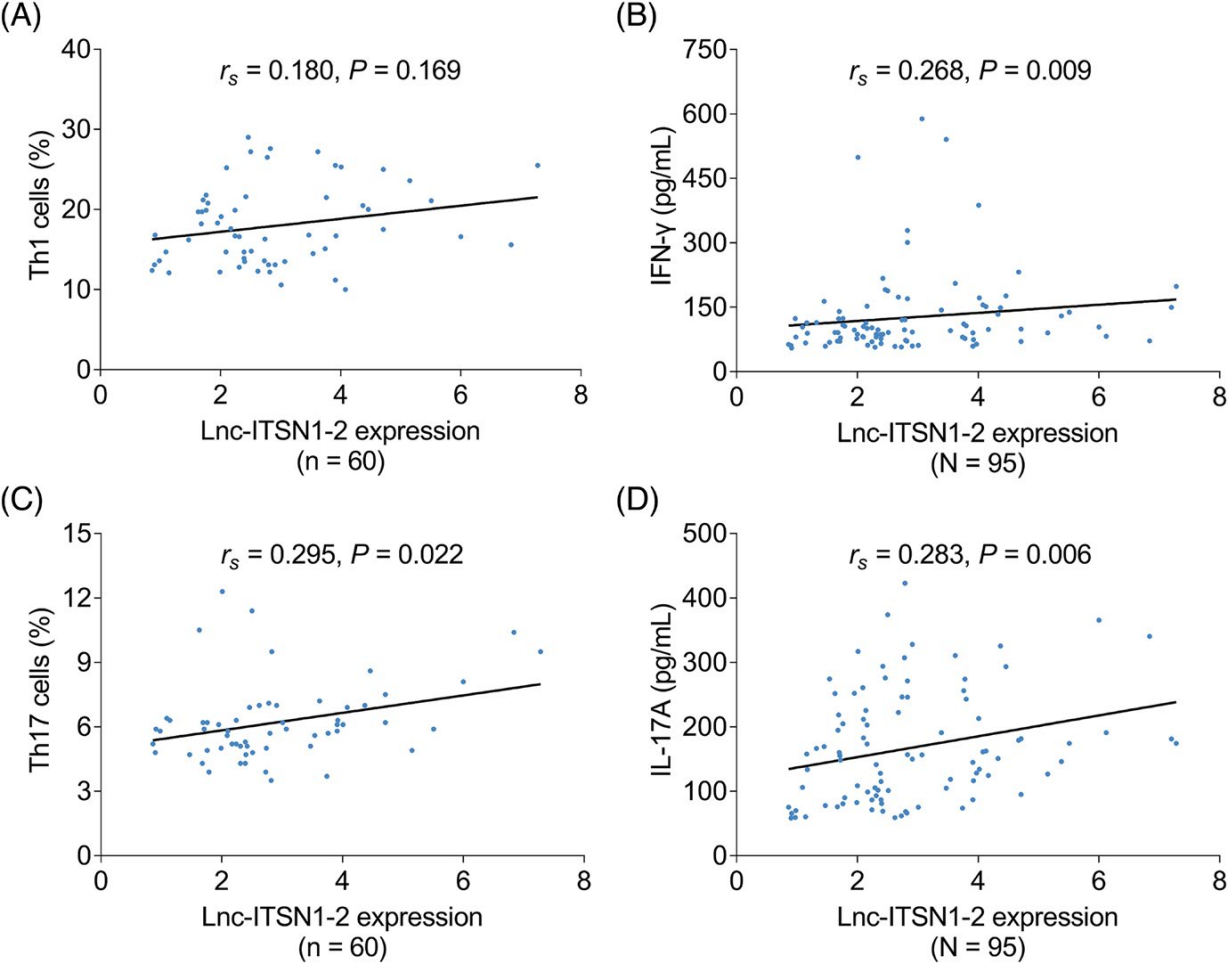
Relationship of lnc‐ITSN1‐2 with Th1 cells and Th17 cells in sepsis patients. Association of lnc‐ITSN1‐2 with Th1 cells (A), IFN‐γ (B), Th17 cells (C), or IL‐17A (D)

Lnc‐ITSN1‐2 did not relate to Th1 cells (*r_s_* = 0.180, *p *= 0.169), while lnc‐ITSN1‐2 linked to high IFN‐γ (*r_s_* = 0.268, *p *= 0.009), Th17 cells (*r_s_* = 0.295, *p *= 0.022) and IL‐17A (*r_s_* = 0.283, *p *= 0.006) in sepsis patients (Figure 2A‐D).

### 3.4 Linkage of lnc‐ITSN1‐2 with inflammation and multiple organ dysfunction

**FIGURE 3:**
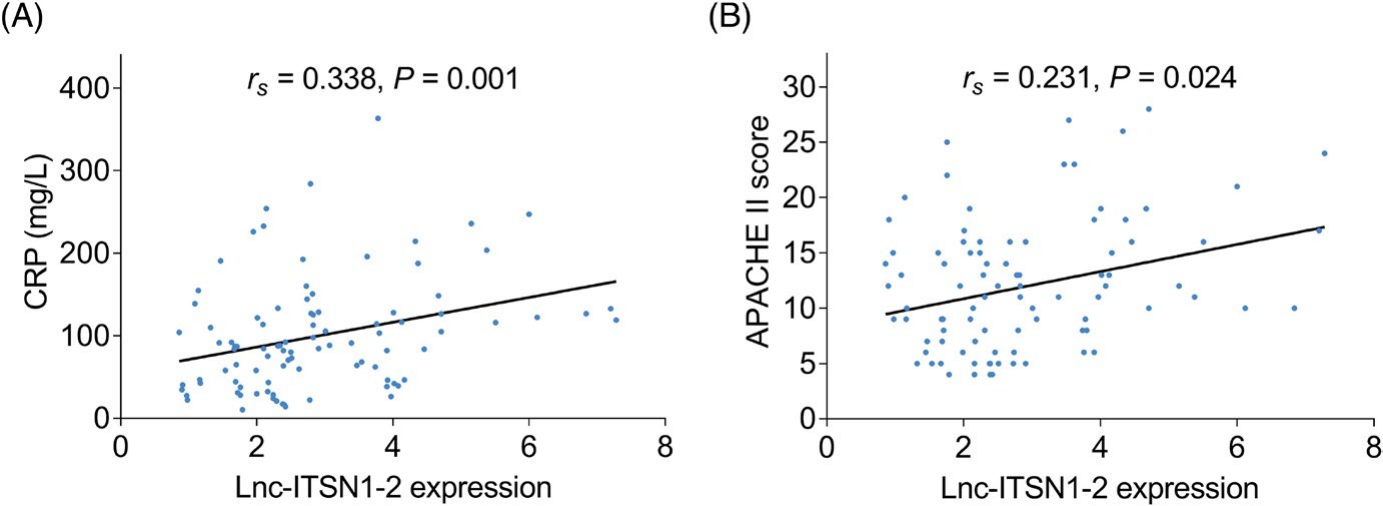
Relationship of lnc‐ITSN1‐2 with inflammation and disease severity in sepsis patients. Association of lnc‐ITSN1‐2 with CRP (A) or APACHE II score (B)

Lnc‐ITSN1‐2 had a positive connection with CRP (*r_s_* = 0.338, *p *= 0.001) and APACHE II score (*r_s_* = 0.231, *p *= 0.024) in sepsis patients (Figure 3A,B). Besides, lnc‐ITSN1‐2 positively associated with total SOFA score (*r_s_* = 0.295, *p* = 0.022) in sepsis patients (Table 2). Regarding SOFA subscales, lnc‐ITSN1‐2 linked to high respiratory system score (*r_s_* = 0.284, *p *= 0.005), cardio vascular system score (*r_s_* = 0.274, *p *= 0.007) and renal system score (*r_s_* = 0.295, *p *= 0.004) (Table 2).

**TABLE 2:** Correlation of lnc‐ITSN1‐2 expression with SOFA score

Items	Correlation coefficient (r_s_)	*p* value
SOFA total score	0.327	0.001
Score of SOFA subscales		
Respiratory system	0.284	0.005
Nervous system	0.113	0.275
Cardio vascular system	0.274	0.007
Liver	0.163	0.114
Coagulation	0.168	0.103
Renal system	0.295	0.004

Abbreviations: lnc‐ITSN1‐2, long non‐coding RNA intersectin 1–2; SOFA, Sequential Organ Failure Assessment.

### 3.5 Linkage of lnc‐ITSN1‐2 with infection sites and organisms

In sepsis patients, lnc‐ITSN1‐2 related to primary infection sites (*Χ* ^2^/*Z* = 8.384, *p* = 0.039); among these, the lnc‐ITSN1‐2 was highest in patients with other infections and lowest in patients with abdominal infection (Table 3). Regarding to primary organisms, lnc‐ITSN1‐2 is only linked to fungus infection (*Χ* ^2^/*Z* = −3.378, *p* = 0.001) (Table 3).

**TABLE 3:** Correlation of lnc‐ITSN1‐2 expression with primary infection site and primary organism

Items	Lnc‐ITSN1‐2 expression Median (IQR)	*Χ^2^*/*Z* value	*P* value
Primary infection site			
Abdominal infection	2.165 (1.675–3.618)	8.384	0.039
Respiratory infection	2.820 (2.150–3.505)		
Skin and soft tissue infection	2.390 (2.000–3.010)		
Other infections	4.370 (2.380–6.120)		
Primary organism			
G‐ bacteria			
No	2.620 (2.120–4.250)	−1.728	0.084
Yes	2.395 (1.750–3.488)		
G+ bacteria			
No	2.560 (1.980–3.828)	−0.279	0.780
Yes	2.390 (1.735–4.035)		
Fungus			
No	2.390 (1.760–3.540)	−3.378	0.001
Yes	4.415 (2.828–6.090)		
Others			
No	2.560 (1.750–3.933)	−0.058	0.954
Yes	2.380 (2.130–3.750)		
Culture negative			
No	2.400 (1.775–3.790)	−0.730	0.466
Yes	2.725 (2.340–3.980)		

Abbreviations: IQR, interquartile range; lnc‐ITSN1‐2, long non‐coding RNA intersectin 1–2.

### 3.6 Comparison of lnc‐ITSN1‐2, Th1, and Th17 cells between deaths and survivors in sepsis patients

**FIGURE 4:**
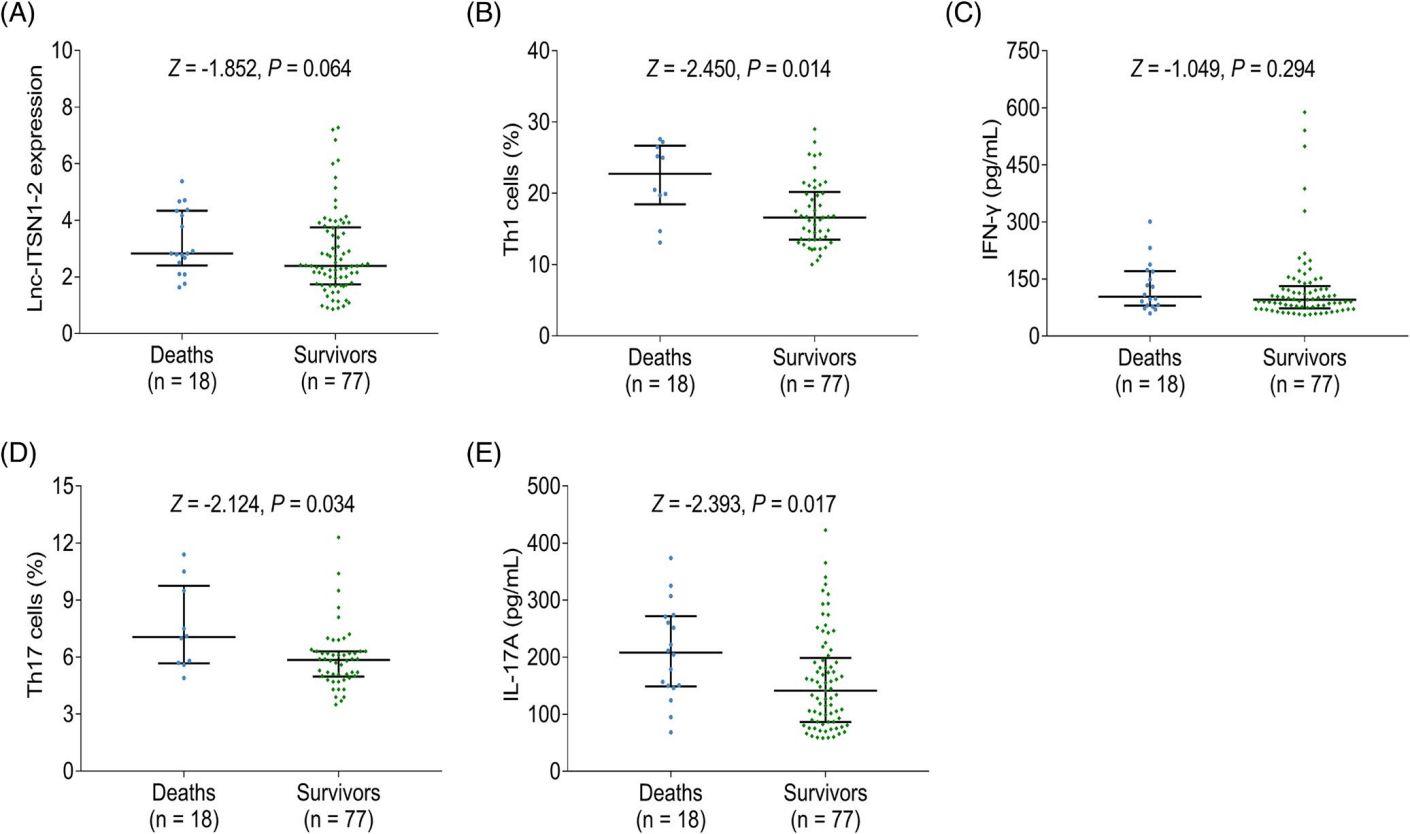
Association of lnc‐ITSN1‐2, Th1 cells, and Th17 cells with mortality risk in sepsis patients. Comparison of lnc‐ITSN1‐2 (A), Th1 cells (B), IFN‐γ level (C), Th17 cells (D), and IL‐17A level (E) between deaths and survivors

Eighteen patients died and the remaining patients survived during the 28 days of follow‐up. Moreover, it was observed that lnc‐ITSN1‐2 (*Z* = −1.852, *p* = 0.064) had an increasing trend in deaths compared to survivors, but did not reach statistical significance (Figure 4A); meanwhile, the Th1 cells (*Z* = −2.450, *p *= 0.014), Th17 cells (*Z* = −2.124, *p *= 0.034) and IL‐17A level (*Z* = −2.393, *p *= 0.017) were enhanced in deaths than those in survivors, but not IFN‐γ level (*Z* = −1.049, *p *= 0.294) (Figure 4B‐E).

Furthermore, univariable logistic regression analysis presented that high lnc‐ITSN1‐2 (odds ratio (OR) = 3.288, *p* = 0.038) was correlated with increased 28‐day mortality; meanwhile, multivariable logistic regression analysis (backward stepwise) illustrated that APACHE II score (OR = 1.135, *p* = 0.009) but not high lnc‐ITSN1‐2 (OR = 3.279, *p* = 0.067) was independently correlated with elevated 28‐day mortality (Supplementary Table 1).

## 4 DISCUSSION

The clinical role of lnc‐ITSN1‐2 in inflammatory‐related or critical ill diseases has drawn wide attention. For instance, a study reports that high lnc‐ITSN1‐2 has been found in ankylosing spondylitis patients, meanwhile, it relates to increased IL‐1β cytokine^25^; another study displays that lnc‐ITSN1‐2 of intestinal mucosa and peripheral blood mononuclear cell in IBD patients is higher than that in health controls^18^; in critical ill diseases, the high expression of lnc‐ITSN1‐2 has been observed in SAP and AIS patients.^16^, ^22^ However, the role of lnc‐ITSN1‐2 in sepsis needs to be further explored. Thus, our research invited 95 sepsis patients and 50 health controls to compare lnc‐ITSN1‐2. Our results displayed lnc‐ITSN1‐2 was upregulated in sepsis patients. The explanations might be that sepsis is characterized by systematic inflammation; meanwhile, lnc‐ITSN1‐2 mediated inflammation by NOD2/RIP2/nuclear factor‐κB (NF‐κB) signaling pathway, which could reflect the level of inflammation to some extent^21^, ^26^; thus, lnc‐ITSN1‐2 was highly expressed in sepsis patients than it in health controls.

Recently, it is reported that lnc‐ITSN1‐2 relates to inflammation and disease severity/activity in critical ill diseases. For example, prior research presents that lnc‐ITSN1‐2 correlates with increased CRP, Ranson's score, APACHE II score in SAP patients^16^; meanwhile, lnc‐ITSN1‐2 positively associates with cognitive impairment, CRP, TNF‐α and IL‐17 levels in AIS patients.^22^ However, the relationship between lnc‐ITSN1‐2 and disease severity in sepsis is not fully understood. In our study, lnc‐ITSN1‐2 was associated with elevated Th17 cells, inflammation, and APACHE II score in sepsis patients. The reason might be that (1) lnc‐ITSN1‐2 might mediate miR‐125a to activate CD4^+^ T cell, induce Th17 cell differentiation, and then release the pro‐inflammatory cytokines^18^; therefore, lnc‐ITSN1‐2 correlated with elevated Th17 cells and inflammation; (2) miR‐125a is a well‐known anti‐inflammatory factor, which inhibits inflammation by regulating the Wnt/β‐catenin and NF‐κB pathways^27^; thus, lnc‐ITSN1‐2 might target miR‐125a (mentioned above) to promote inflammation, thus causing multiple organ injury, which indirectly increased disease severity in sepsis patients.

Besides, our study presented the relationship between lnc‐ITSN1‐2 and multiple organ dysfunction in sepsis patients, which showed that the lnc‐ITSN1‐2 related to raised SOFA score; moreover, it was positively associated with respiratory system, cardiovascular system, and renal system injury. The data might be caused by that (1) high lnc‐ITSN1‐2 regulated the inflammation (described above), which accelerated multiple organ injury in sepsis patients; (2) lnc‐ITSN1‐2 might facilitate cell injury and apoptosis,^28^ which directly induced the multiple organ injury.

At present, lnc‐ITSN1‐2 as a prognostic biomarker for critical ill diseases has been illustrated such as AIS and SAP.^16^, ^22^ For example, lnc‐ITSN1‐2 has a connection with worse recurrence‐free survival in AIS patients^22^; regarding SAP, lnc‐ITSN1‐2 in deaths is higher than that in survivors; meanwhile, it discloses decent predictive value for elevated mortality risk.^16^ In the present study, we evaluated the relationship of lnc‐ITSN1‐2 with prognosis in sepsis patients, which illustrated that lnc‐ITSN1‐2 presented an increasing trend in deaths. The possible explanation was listed as follows: (1) lnc‐ITSN1‐2 had a connection with high inflammation, APACHE II score, SOFA score, which led to high mortality risk; (2) the sample size of this study was relatively small; therefore, lnc‐ITSN1‐2 only exhibited an increasing trend, but no statistical difference in deaths compared to survivors.

The current study was cross‐sectional research, which revealed that lnc‐ITSN1‐2 might be a potential biomarker of sepsis, while the causality between sepsis and lnc‐ITSN1‐2 was hard to explore. Some limitations still existed in the current research: (1) the sample size of sepsis patients was only 95, which might affect the power in statistics, thus, subsequent study should involve more sample size to verify our conclusion; (2) the mechanism of lnc‐ITSN1‐2 facilitating Th17 cell differentiation and multiple organ dysfunction in sepsis was not clear, which should be explored in the following study; (3) the change of lnc‐ITSN1‐2 in sepsis patients was unclear after recovery, which should be explored in following study; (4) level of lnc‐ITSN1‐2 in urine and tissue apart from PBMC in sepsis patients could be investigated; (5) the quantification of lnc‐ITSN1‐2 was relative in the current study, the absolute quantification could be explored in future; (6) the level of lnc‐ITSN1‐2 in septic shock could be explored in the further study; (7) whether lnc‐ITSN1‐2 was variated by treatment regimens such as antibiotics, anti‐inflammation in sepsis could be explored in future.

In conclusion, lnc‐ITSN1‐2 is highly expressed and correlates with inflammation, multiple organ dysfunction, and mortality risk in sepsis patients, indicating lnc‐ITSN1‐2 may serve as a potential biomarker in sepsis patients.

## CONFLICT OF INTEREST

The authors declare that they have no conflicts of interest.

## Supporting information

Table S1

## Data Availability

The datasets generated during and/or analyzed during the current study are available from the corresponding author on reasonable request.
