# Supplementary material for: Blood long non‐coding RNA intersectin 1–2 is highly expressed and links with increased Th17 cells, inflammation, multiple organ dysfunction, and mortality risk in sepsis patients
Source: J Clin Lab Anal. 2022 Mar 4;36(4):e24330. doi: 10.1002/jcla.24330 (PMC8993609; doi:10.1002/jcla.24330)
Supplement: Supplementary file 1 — Table S1 [file JCLA-36-e24330-s001.docx]

**Supplementary Table 1.** Logistic regression analysis for 28-day mortality.

| Items | *P* value | OR | 95% CI | |
| --- | --- | --- | --- | --- |
|  |  |  | Lower | Upper |
| **Univariable logistic regression analysis** |  |  |  |  |
| Lnc-ITSN1-2 expression (high vs. low) | **0.038** | 3.288 | 1.067 | 10.131 |
| Age (years) | 0.299 | 1.024 | 0.979 | 1.070 |
| Gender (male vs. female) | 0.493 | 1.486 | 0.479 | 4.604 |
| BMI (kg/m^2^) | 0.556 | 1.486 | 0.479 | 4.604 |
| Smoke (yes vs. no) | 0.915 | 0.944 | 0.330 | 2.702 |
| Drink (yes vs. no) | 0.140 | 2.187 | 0.774 | 6.184 |
| History of hypertension (yes vs. no) | 0.923 | 1.053 | 0.367 | 3.021 |
| History of hyperlipidemia (yes vs. no) | 0.500 | 1.548 | 0.434 | 5.513 |
| History of diabetes (yes vs. no) | 0.410 | 1.714 | 0.476 | 6.175 |
| History of CKD (yes vs. no) | 0.650 | 1.479 | 0.273 | 8.014 |
| History of CCVD (yes vs. no) | 0.827 | 0.872 | 0.256 | 2.972 |
| Primary infection site |  |  |  |  |
| Other infections | Reference |  |  |  |
| Abdominal infection | 0.375 | 0.509 | 0.115 | 2.260 |
| Respiratory infection | 0.850 | 0.868 | 0.200 | 3.766 |
| Skin and soft tissue infection | 0.298 | 0.413 | 0.078 | 2.187 |
| Primary organism (culture negative vs. G- bacteria, G+ bacteria, fungus or others) | 0.093 | 2.906 | 0.838 | 10.080 |
| CRP (mg/L) | **0.024** | 1.008 | 1.001 | 1.016 |
| APACHE II score | **0.004** | 1.141 | 1.043 | 1.248 |
| SOFA score | **0.002** | 1.421 | 1.138 | 1.775 |
| **Multivariable logistic regression analysis (backward stepwise)** | | |  |  |
| Lnc-ITSN1-2 expression (high vs. low) | 0.067 | 3.279 | 0.918 | 11.703 |
| Age (years) | 0.105 | 1.046 | 0.991 | 1.104 |
| APACHE II score | **0.009** | 1.135 | 1.033 | 1.248 |

OR, odds ratio; CI, confidence interval; lnc-ITSN1-2, long noncoding RNA intersectin 1-2; BMI, body mass index; CKD, chronic kidney disease; CCVD, cerebrovascular and cardiovascular diseases; CRP, C-reactive protein; APACHE II, Acute Physiology and Chronic Health Evaluation II; SOFA, Sequential Organ Failure Assessment.
